# FragTracer: Real-Time Fragmentation Monitoring Tool for F2FS File System

**DOI:** 10.3390/s23094488

**Published:** 2023-05-05

**Authors:** Minseon Cho, Donghyun Kang

**Affiliations:** 1 Department of Computer Engineering, Changwon National University, Changwon 51140, Republic of Korea; 2 Department of Computer Engineering, Gachon University, Seongnam 13120, Republic of Korea

**Keywords:** fragmentation, file systems, database systems, monitoring, visualization, tool

## Abstract

Emerging hardware devices (e.g., NVMe SSD, RISC-V, etc.) open new opportunities for improving the overall performance of computer systems. In addition, the applications try to fully utilize hardware resources to keep up with those improvements. However, these trends can cause significant file system overheads (i.e., fragmentation issues). In this paper, we first study the reason for the fragmentation issues on an F2FS file system and present a new tool, called FragTracer, which helps to analyze the ratio of fragmentation in real-time. For user-friendly usage, we designed FragTracer with three main modules, monitoring, pre-processing, and visualization, which automatically runs without any user intervention. We also optimized FragTracer in terms of performance to hide its overhead in tracking and analyzing fragmentation issues on-the-fly. We evaluated FragTracer with three real-world databases on the F2FS file system, so as to study the fragmentation characteristics caused by databases, and we compared the overhead of FragTracer. Our evaluation results clearly show that the overhead of FragTracer is negligible when running on commodity computing environments.

## 1. Introduction

There are a lot of valuable studies on how to improve the overall performance of computer systems [1,2,3,4,5,6,7,8,9,10,11,12]. For example, since the overall performance depends on the latency of input/output operations, many researchers in academia and industry have focused on hardware improvements (e.g., non-volatile memory, CXL, and NVMe SSD devices). Intel introduced a new storage device, DCPMM, that is 10 times faster compared with solid state drives (SSDs) [13,14,15]. Other researchers deep dive into the traditional software stacks (e.g., page cache, file system, and block layer) because the stacks are struggling to keep up with the hardware improvements. Especially, much effort has been dedicated to a file system that can drop its overall performance when it requires extra operations; (1) it performs extra write to guarantee the crash consistency of data (i.e., journaling or logging) and (2) it sometimes performs extra I/O operations to split or merge blocks [16,17,18,19,20,21]. Unfortunately, those extra operations lead to a high I/O latency by amplifying the amount of writes and making the file system fragmented. In addition, the file system becomes further fragmented over time as more applications issue multiple I/O operations [11,22]. To solve such a fragmentation issue, some researchers have proposed defragmentation tools that diagnose the data in the file system and that make up free space consecutively [9,23,24]. However, the results of prior studies are unable to be easily used in all kinds of operating systems or applications because they have been implemented in the kernel layer.

In this paper, we propose a user-layer tool called FragTracer, which diagnoses the ratio of fragmentation in the file system, based on analyzing the patterns of I/O operations issued by applications on-the-fly. For user-friendly usage, we designed FragTracer, which is composed of three major modules, the monitoring, pre-processing, and visualization modules. The monitoring module periodically collects internal information on the file system; it describes where the application’s data are allocated and freed. The pre-processing module reconstructs the collected information for the next step because the collected information includes unnecessary information for detecting fragmentation issues. To help in directly understanding the ratio of the fragmentation, the visualization module shows the file system fragmentation in the form of a color table. We implemented FragTracer and evaluated it based on a log-structured file system (i.e., F2FS [5]) with three database applications to confirm that the fragmentation issue is not closed yet, even with a sequential pattern file system and database applications. In addition, we measured the runtime overhead of FragTracer that simultaneously and independently runs for monitoring the ratio of current fragmentation. As a result, we determined that FragTracer increases the total runtime of the database application by up to 21% compared with the baseline.

The contributions of this work are as follows:

- We study the fragmentation issue in file systems and describe why the used space is fragmented using the ftruncate system call, which is widely used to free up space.
- We propose FragTracer, a tool to automatically diagnose fragmented blocks on the file system in real-time without any modification in their kernel layer.
- We evaluate FragTracer on an F2FS file system with three major database applications, such as RocksDB [25], Redis [26], and VoltDB [27].

The remainder of the paper is composed as follows. Section 2 presents the fragmentation issue in file systems and previous studies to overcome the issue. In Section 3, we introduce the design of FragTracer in detail. Section 4 explains our experimental setup and shows our evaluation results based on an F2FS file system with three database applications. Lastly, we draw up the conclusions in Section 5.

## 2. Background and Motivation

In this section, we briefly review software approaches that perform sequential access for high performance and low fragmentation, and then describe our motivation.

### 2.1. F2FS File System and Fragmentation

File systems have been considered a crucial part in computing systems because they are responsible for the mapping between I/O requests from applications and the space of the storage media [22,28]. Therefore, there were significant efforts in designing file systems that aim to enhance the characteristics of storage media while handling concurrent I/O requests from applications [3,4,5,6,11,12]. For example, the F2FS file system was designed for flash-based storage media (e.g., SSD and NVMe SSD) where the performance of sequential access patterns is faster than that of random patterns by an order of magnitude; it transforms random I/O requests by applications into sequential ones [5,8]. However, the F2FS file system suffers from the fragmentation issue that occurs when using the file systems for a long time. The major reason for the issue is that files are frequently created and removed, and their size is variable. In addition, applications call the ftruncate system call to resize the file created previously; they trigger ftruncate when data belonging to the file are no longer used, or when pre-allocated blocks using a fallocate system call are unnecessary [28,29]. However, a frequent ftruncate call accelerates the fragmentation issue because of its following mechanism. First, an application calls the ftruncate system call with a file descriptor and offset to shrink the file size. Then, the file system finds the metadata of the corresponding file and updates the information of the size based on the received offset. Unfortunately, at this time, the shrunk region may not be aligned with a page unit (i.e., 4096 bytes) because the offset denotes the size in bytes. Therefore, the file system has to invalidate the region that is no longer used if the offset is not divisible by 4096 bytes. Such an invalidation leads to moving the corresponding block into another segment; F2FS follows the basic rule of a log-structured file system. As a result, the file based on sequential LBAs is split into two or more due to ftruncate. When the application accesses the partitioned file, the file system issues more I/O operations to the underlying storage device, in that the I/O requests cannot be merged. Thus, it delays the latency of I/O requests to the file.

On the other hand, some researchers have focused on solving the fragmentation issue and have proposed several tools for monitoring file system fragmentation, such as *filefrag* and *f2fs_io* [30,31]. *Filefrag* [30] is a file system fragmentation monitoring tool developed for Ext file systems (e.g., Ext3 and Ext4); it returns the extent information of the file through FIEMAP or FIBMAP I/O control (IOCTL) system calls. *Filefrag* returns information about the file extent; the extent refers to a set of data blocks of a file allocated to a continuous space. The number of extents belonging to a file indicates whether it is fragmented. Meanwhile, f2fs_io [31] is an IOCTL tool developed for the F2FS file system, and it contains the function of returning the locations of data blocks. With these monitoring tools, information about the file system fragmentation of the files can be obtained. However, these tools do not support real-time monitoring, which allows for the continuous analysis of file I/O patterns.

### 2.2. In-Memory Database System

Nowadays, in-memory database systems (i.e., key-value stores) are more popular and becoming flash-friendly, which enhances the performance and endurance of NAND-flash storage devices by processing their data sequentially. The reason for this is that the database system has high opportunities for scalability and availability, in that it can handle any type of data in a key-value pair [32]. For example, RocksDB is widely adopted in data centers to handle large data management [25]. It employs sorted sequence table (SST) files and write-ahead log (WAL) files to use a way for all data to be issued to the underlying storage device in sequential and bulk patterns (i.e., flash-friendly); SST files store database records, while WAL files are log files used to prevent the loss of record data that have not yet been stored in SST files. Redis and VoltDB are also key-value stores using the snapshot mechanism [26,27]. Redis provides the Redis database (RDB) and append-only file (AOF) for guaranteeing its data consistency, and it periodically makes and stores a snapshot of the entire database on the storage media. VoltDB utilizes a snapshot mechanism for logging the commands processed. In summary, in-memory database systems tend to prefer sequential and bulk writes. Therefore, many users think that the key-value stores can alleviate the fragmentation issue of the file system.

### 2.3. Motivation

As mentioned before, the pattern of sequential access in software is becoming a new trend by widely adopting high-performance storage media (e.g., DCPMM, NVMe SSD, and SSD) as storage or a cache layer [13,14,15]. FragTracer is motivated by the question: *Can we free the fragmentation issue if we use both a sequential file system and a sequential application?* In other words, FragTracer helps to identify the fragmentation issue with ease.

## 3. Design of FragTracer

In this section, we propose a new monitoring tool, called FragTracer, that collects I/O patterns issued from the file system and identifies the fragmentation ratio in a visible form. Especially, FragTracer focuses on how data are placed in the file system because the fragmentation issue comes from the placement caused by the write operations. To isolate the steps of FragTracer, we organized FragTracer into three modules: monitoring, pre-processing, and visualization. For real-time identification, the monitoring module collects write operations that are passed to the file system layer. Then, the pre-processing module scans the collected data and determines valuable data correlated to the fragmentation issue. Finally, the visualization module is responsible for the visualization of the fragmented units belonging to the file system, for the user watching of fragmentation with ease. Unfortunately, it is challenging to identify the fragmentation of the file system on-the-fly due to concentrated I/O requests issued from lots of running applications. In addition, the visualization modules may interfere with the performance of running applications because it also requires hardware resources, such as CPU and memory. To mitigate the performance interference, we designed that the monitoring module and pre-processing module are performed using a background thread. The background thread is allocated for each monitoring path and it is triggered based on the pre-defined interval. We design FragTracer to periodically collect (i.e., monitoring module) and identify fragment units (i.e., pre-processing module) using background threads. Meanwhile, the visualization module is triggered by user requirements and it offloads lots of I/O requests into the background thread. Algorithm 1 shows the behaviors of FragTracer as a pseudo-code.
**Algorithm 1:** Real-time monitoring of FragTracer  **Input:**
*p*: monitoring directory path, *T_i_*: monitoring interval     1: **module** MonitoringModule(*p*):     2: monitoringTarget ← TargetDetermination(*p*)     3: monitoringResult ← filefrag(monitoringTarget)     4: **return** monitoringResult     5:      6: **module** Pre-processingModule(fragInfo.txt):     7: extractedInfo ← Extraction(fragInfo.txt)     8: writtenExt ← ExcludeUnwritten(extractedInfo)     9: sortedExt ← sorted(writtenExt)    10:   **return** sortedExt    11:      12: **function** RealTimeMonitoring(*p*):    13:   fragInfo.txt ← MonitoringModule(*p*)    14:   fragProc.tbl ← Pre-processingModule(fragInfo.txt)    15:   **return**    16:      17: **while** true **do**    18:  RealTimeMonitoring(*p*) &    19:  Sleep($T_{i}$)


### 3.1. Monitoring Module

The monitoring module focuses on the organization of a file that may affect fragmentation in the file system. However, it is a key challenge to monitor the organization of a file on-the-fly because it can be frequently updated. To overcome such a challenge, the monitoring module utilizes the fact that many applications tend to place their own files in the same directory; most applications perform file I/O for file creation, modification, and deletion commands within a particular directory. In other words, this module monitors the organization of each file based on the directory in real-time. To collect valuable information, the monitoring module performs the following steps. (1) This module is executed together with the directory, which means the monitoring path for collecting the organization of files (line 3 in Algorithm 1); the monitoring module targets all existing files belonging to the path and collects information about them. (2) After the target path is determined, this module periodically triggers *filefrag* to collect the extent information of each target file (line 4 in Algorithm 1); *filefrag* helps to gather the information of extent, regardless of the file system types (e.g., Ext3, Ext4, and F2FS). (3) Since the collected extents can give a hint to determine whether a file is fragmented or not on the file system, this module creates “fragInfo.txt” on a per-directory basis and stores the extent information, respectively (line 13 in Algorithm 1).

### 3.2. Pre-Processing Module

**Figure 1:**
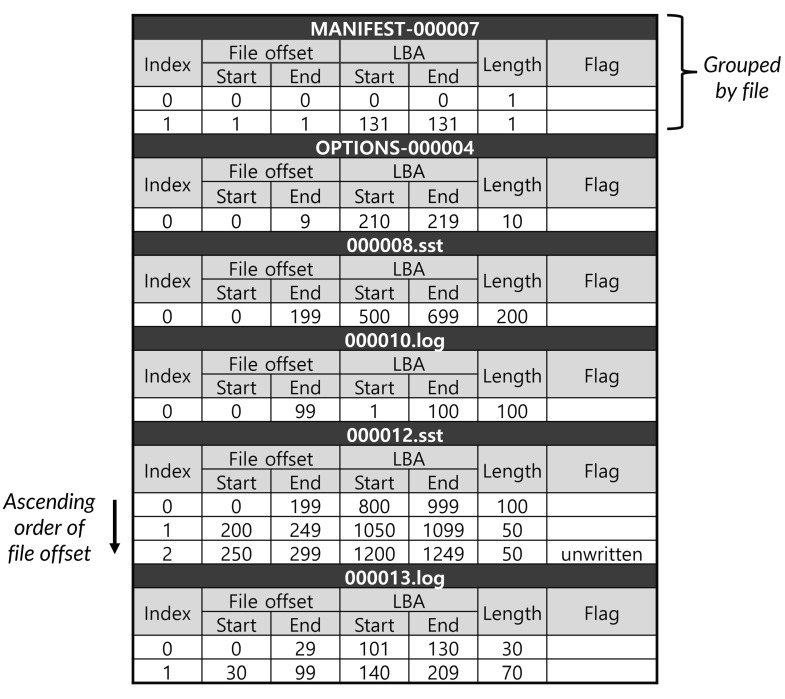
Example of fragInfo.txt.

**Figure 2:**
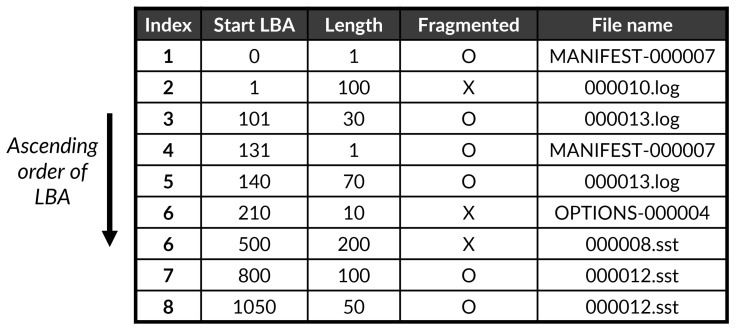
Example of fragProc.tbl.

Figure 1 shows the information of the collected extent from the monitoring module. As shown in Figure 1, each extent contains the following information: file name, start offset, end offset, start LBA, end LBA, length, and flags. The pre-processing module filters out the necessary information in “fragInfo.txt” file to determine whether a file is fragmented or not: fine name, start LBA address of an extent, length of the extent, and flag (line 7 in Algorithm 1). The key information is the length because it means that the corresponding extent is contiguously placed from the start LBA address on the file system. In general, a file can be composed of one or more extents and the location of each extent in the file system depends on the sequence and timing of the write operations. If a file includes multiple extents and their LBA addresses are non-contiguous on the file system, it means that the file was fragmented with multiple extents. The higher the number of non-contiguous extents in a file, the worse the fragmentation issue that causes the collapse of the overall performance for the file access. Thus, the role of the pre-processing module is to scan extents that belong to the same file and to find non-contiguous ones. This scanning procedure skips extents whose “unwritten flag” is set as 1 (line 8 in Algorithm 1). The reason for such a skip is that the extent has not been permanently written yet on the file system; modern file systems (e.g., F2FS, ext4, and xfs) employ the *delayed allocation* that postpones allocating data in physical data blocks for performance improvement and optimization [33]. For example, an extent (index #2) belonging to the 000012.sst file in Figure 1 is skipped due to the behavior of the *delayed allocation*. Finally, the pre-processing module sorts the collected information from the scanning procedure based on the start LBA address (line 9 in Algorithm 1) and then stores the sorted information in the “fragProc.tbl” (see Figure 2).

### 3.3. Visualization Module

**Figure 3:**
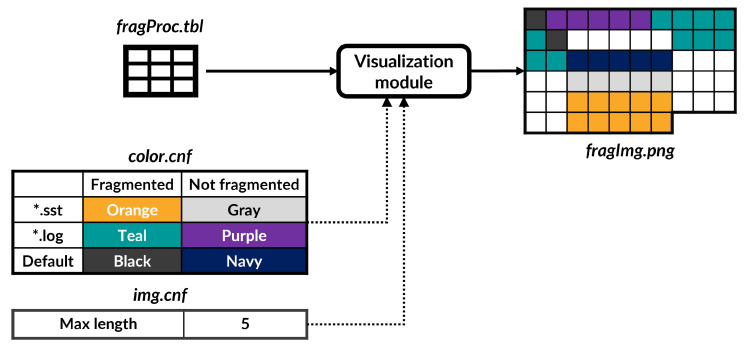
Visualization module.

This module is responsible for visualizing the ratio of fragmentation on the file system with the information transformed from the pre-processing module (i.e., fragProc.tbl file). Figure 3 shows the overall process of this module when a user executes this module. As shown in Figure 3, this module makes a “fragImg.png” file as a result, with two configuration files: “color.cnf” and “img.cnf”. The “fragImg.png” file is composed of boxes, and each box means one logical block on the file system. The “color.cnf” file identifies fragmented areas based on the extension of files to clearly display the placement of data. In other words, the file maps fragmented and non-fragmented areas with different colors, as shown in Figure 3. The “img.cnf” file includes the maximum length to accelerate visualizing performance. Making the “fragImg.png” file requires high amounts of hardware resources and a lot of time, and the part written as a sequential region is unnecessary to display to a user. Therefore, we define the sequential region based on the number of contiguous LBA addresses. If the number of contiguous LBAs exceeds the maximum length, the visualization module does not record that region in the “fragImg.png” file. Otherwise, this module records the fragmented extents according to the color listed in the “color.cnf”. For example, if an extent includes non-contiguous LBA addresses, the data blocks belonging to the file titled with the “.sst” extension are displayed with an orange color (see “fragImg.png” in Figure 3). Therefore, a user easily discovers which file is fragmented or not, using the “fragImg.png” file, and can utilize such information to optimize the performance of the file system.

## 4. Evaluation

In this section, we describe our experimental setup in detail and answer the following two questions:

- How does FragTracer perform on a Log-structured file system with key-value database applications?
- What are the performance overheads of FragTracer?

### 4.1. Experimental Setup

To confirm the functionality of FragTracer, we implemented FragTracer with an existing command (i.e., *filefrag*) that reports the level of fragmentation for a particular file. Thus, it is worth noting that FragTracer can be used without any modification of the kernel or at the applications-level; users can use FragTracer on any file system and application with ease. We performed our experiments on the real machine with Intel i9-12900 KF, 32 GiB memory, and a 256 GB Samsung SSD (see Table 1). We ran all our experiments on an F2FS file system, which handles data in a way that optimizes fragmentation by performing sequential writes. To confirm the effectiveness of FragTracer, we also used three different database applications: RocksDB, Redis, and VoltDB. RocksDB is a key-value store that sequentially processes its data with bulk write operations. Redis is a NoSQL database system that guarantees low latency using the memory region as its cache for data. VoltDB is a NewSQL database system that also uses the main memory for data storage. In summary, three database applications temporally cache data and objects in the memory region to speed up the overall performance.

### 4.2. The YCSB Workload

We used the YCSB (Yahoo! Cloud Serving Benchmark), which is widely used for the experiments of file systems because it can generate various I/O workloads with the Zipf distribution. Since the fragmentation level is closely related to the writing pattern, we ran the workload-F, which makes read and read-modify-write operations at a ratio of 5:5 (see Table 2). This workload runs over 5 h and its execution time highly depends on the database. Therefore, to understand the difference in terms of the level of fragmentation, we took a snapshot of the data blocks after 60 s and 1 h while replaying the workload on the three databases.

#### 4.2.1. RocksDB

As mentioned before, we first set the color information in the ’color.cnf’ file to identify the fragmented blocks. Since RocksDB [25] employs three different extension types of files for high performance and for guaranteeing data consistency, we configured each type in different colors to find fragmented units. Table 3 describes how to map each file extension to color; we defined two different colors in the fragmented blocks to take turns presenting the colors for the fragmented blocks.

**Figure 4:**
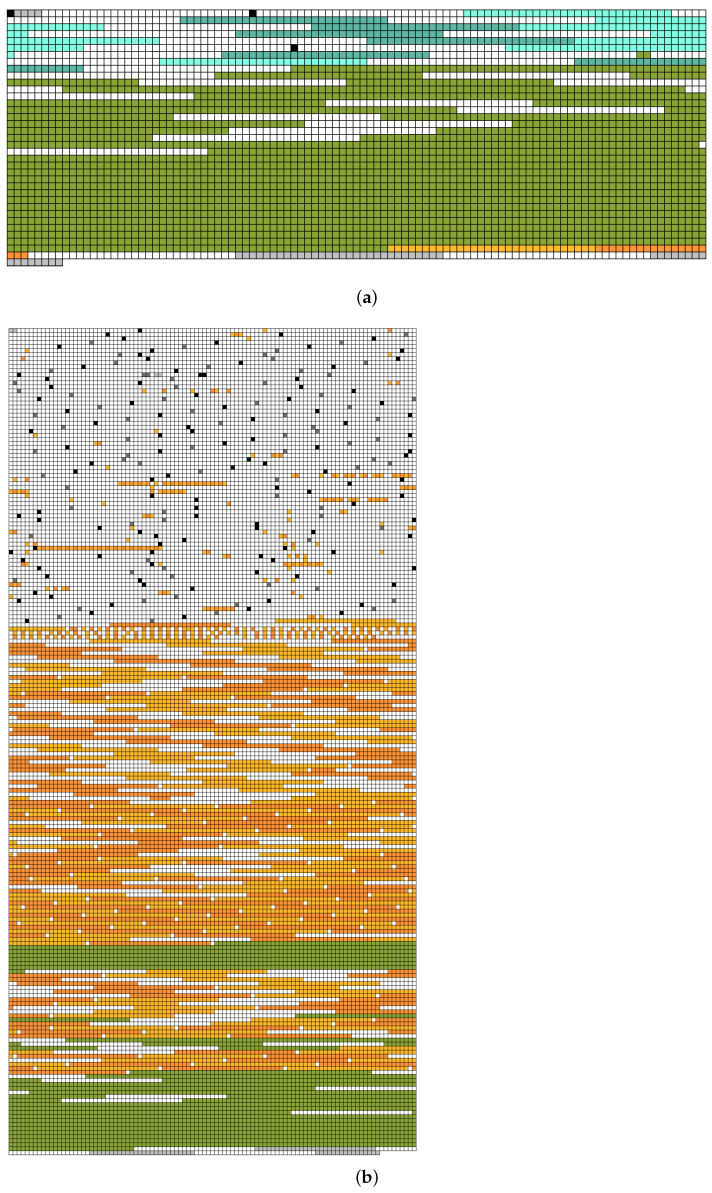
Visualization of file system fragmentation of RocksDB; (**a**) The snapshot after 60 s; (**b**) The snapshot after 1 h.

Figure 4 presents the results of FragTracer; the empty space was filled in white color. To clearly understand the level of fragmentation, we first ran FragTracer, which collects the information of data blocks at 60 s while running workload-F (see Figure 4a). As shown in Figure 4a, most of the squares are filled with green color; this color means that blocks are used for saving the data of .sst files. As we expected, most of the blocks belonging to the .sst files are placed in a sequential way without any fragmentation. There are two major reasons behind this. (1) RocksDB was designed to be flash-friendly; thus, it uses a sequential and bulk write. (2) Since the time for monitoring is very short, the same blocks were not frequently updated. In this case, the F2FS file system never performs in-place updates (i.e., new data are written to another place, not the original place). To investigate the results of the long-term test, we performed the same evaluation and monitored it after 1 h (see Figure 4b). Surprisingly, the pattern as shown in Figure 4b is totally different compared to that of Figure 4a. First of all, the number of squares with orange color is much increased; this color means that blocks belonging to the .sst files are fragmented. We deep-dived into the reason for such a difference and figured out that the last block of each .sst file is not placed with contiguous LBAs. Such a pattern is caused by the RocksDB, where a set of system calls is employed to guarantee crash consistency (e.g., fdatasync) and to secure contiguous space on the file system (i.e., fallocate and ftruncate). For example, RocksDB calls fallocate to allocate space in advance. It issues fdatasync so as to permanently store data in in-memory into the underlying storage media. Finally, RocksDB triggers ftruncate to discard space that is not used. Meanwhile, the F2FS file system handles the system calls issued from the RocksDB and allocates the location of the last block twice. (1) The first location of the last block is set by fdatasync with the contiguous block. (2) When calling ftruncate, the location becomes invalid and the new location is allocated for the last block.

In summary, RocksDB on an F2FS file system is a very attractive database if small update operations occur for a short time; however, it suffers from the fragmentation issue over time.

#### 4.2.2. Redis

Redis [26] is one of the database applications and it utilizes in-memory RDB (Redis database) with the snapshot approach of the database. In Redis, all data stored in RDB are handled in the main memory, and the consistency of RDB is guaranteed by writing all states of RDB in the form of a binary snapshot. Table 4 shows the mapping information between the extension of files and color; we defined two different colors in the fragmented region to take turns presenting color for the fragmented blocks.

**Figure 5:**
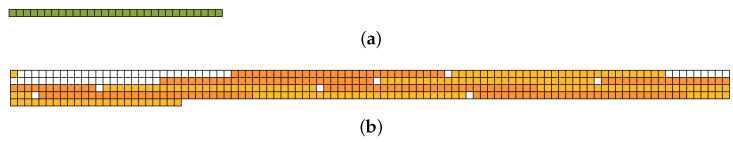
Visualization of file system fragmentation of Redis; (**a**) The snapshot after 60 s; (**b**) The snapshot after 1 h.

Figure 5 shows our evaluation results of Redis. Even though all configurations were set to be the same as RocksDB, Figure 5 shows different results compared with that of RocksDB.

First of all, the amount of write operations is significantly small, in that Redis rarely issues write operations to store a snapshot version of the RDB. As a result, Figure 5a and Figure 5b show a very small set of squares compared with those of RocksDB, even though tests ran after 60 s or 1 h, respectively. As shown in Figure 5a, Redis never reveals the fragmentation issue on the test for 60 s due to the same reason as RocksDB. However, in the long-term test, Redis also suffers from the issue; the orange color means that blocks belonging to the .rdb file were fragmented over time (see Figure 5b). To understand the reason, we monitored the data blocks used for each .rdb file using strace system call [34]. We figured out the fact that some blocks, including empty data, disconnect the neighborhood of LBA blocks. The empty blocks were generated using update operations with fdatasync to consistently store data belonging to the last block of each .rdb file. In summary, Redis evidently suffers from a fragmentation issue over time because of the update for the last block, even though it performs the best policies for making sequential writes.

#### 4.2.3. VoltDB

We also employed VoltDB [27] as a database application on the F2FS file system. Since VoltDB uses four different types of file extensions, such as “.vpt”, “.digest”, “.hash”, and default files, we configured the mapping information between the extension of files and color as shown in Table 5; we defined two different colors in the fragmented state to take turns presenting the color for the fragmented blocks.

**Figure 6:**
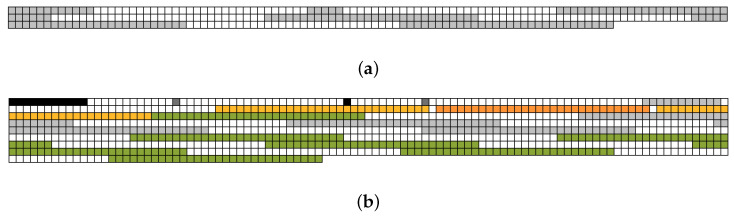
Visualization of file system fragmentation of VoltDB; (**a**) The snapshot after 60 s; (**b**) The snapshot after 1 h.

Figure 6 shows our experimental results that come from the visualization module. VoltDB also uses a snapshot mechanism to guarantee the consistency of data. In our experiment, the period of the snapshot is set as 30 min; thus, a database file is created every 30 min. Figure 6a shows the ratio of fragmentation after running VoltDB over 60 s. As expected, Figure 6a only presents blocks colored with light gray because the snapshot is never triggered; this means that files are not fragmented, and the “.vpt”, “.digest”, and “.hash” files are used for the snapshot mechanism. Unlike Figure 6a, Figure 6b shows boxes with various colors because we collected them after running VoltDB for 1 h; in this case, the snapshot for guaranteeing the consistency of data is twice called during the evaluation. Figure 6b clearly shows that VoltDB also reveals the fragmentation issue over time; it presents two different orange colors. To study the reason for such fragmentation in the “.vpt” file, we monitored the system calls issued from VoltDB using strace. We figured out VoltDB calls write operations, along with SYNC_FILE_RANGE, for handling the snapshot mechanism, and the size of each operation is sometimes smaller than one page (i.e., 4096 bytes).

### 4.3. Overhead of FragTracer

To understand the overhead of FragTracer, we measured the execution time while running a database application along with FragTracer, and compared it with the baseline, which only runs a database application.

**Figure 7:**
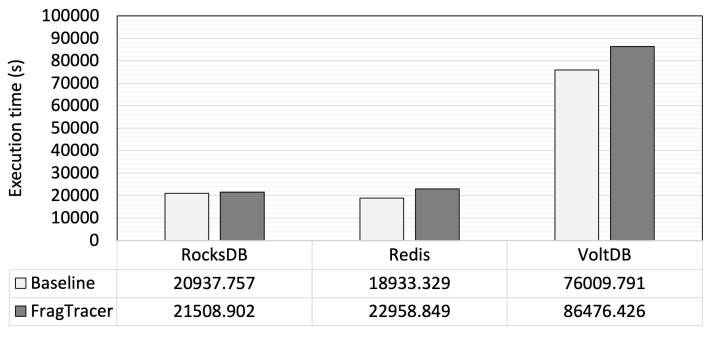
The total execution time.

Figure 7 shows the total execution time when workload-F is executed on different databases, respectively. As shown in Figure 7, RocksDB and Redis show a similar execution time whether FragTracer runs or not; FragTracer includes only a 2% runtime overhead compared with the baseline. The results are interesting in that FragTracer indeed uses the hardware resources (e.g., CPU, memory, and I/O block) for monitoring the ratio of fragmentation and generating it in graphical form. Meanwhile, VoltDB shows a larger overhead than other database applications. In VoltDB, the FragTracer increases the runtime by 21% compared with the baseline. We believe that it is a reasonable overhead, in that FragTracer shows the ratio of fragmentation with ease and that it can sometimes be utilized for analyzing the performance issue on the filesystem.

**Figure 8:**
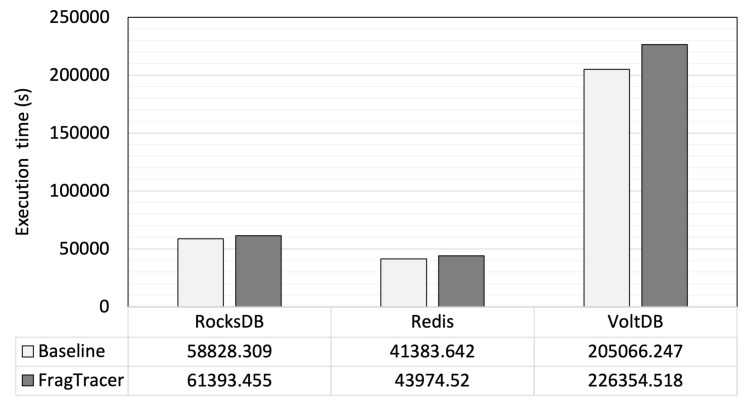
The total execution time with an I/O-intensive workload.

Additionally, we repeated the above experiments under an I/O-intensive condition to simulate the scenario with a heavy I/O workload. We measured the application runtime with intensive random I/O on the same storage as the file I/O of the application using the fio [35]. The fio workload settings are presented in Table 6. Figure 8 shows the results of the evaluation. The runtimes of the applications monitored by FragTracer increased by up to 10% compared with the runtimes of the applications without the monitoring of FragTracer. The differences between the base runtime and the runtime with FragTracer monitoring were smaller than those shown in Figure 7. It can be considered that massive I/O has a greater impact on the application performance; therefore, the impact of FragTracer seems relatively small in an I/O-intensive condition.

## 5. Conclusions

In this paper, we proposed FragTracer, which is a monitoring tool for analyzing the file I/O patterns of applications. Since file fragmentation reflects the file I/O pattern, FragTracer monitors and visualizes file system fragmentation in real-time. To evaluate FragTracer, we monitored RocksDB, Redis, and VoltDB using FragTracer in real-time. The file I/Os of the applications can be analyzed using images generated through real-time monitoring. Additionally, the application runtime with FragTracer monitoring increased by up to 21% compared with the base runtime of each application. In the I/O-intensive condition, the application runtime with FragTracer monitoring increased by up to 10% compared with the base runtime of each application. The results confirm that FragTracer does not have a significant impact on the base runtime of each application, and that these factors affect the performance of an application.

## Figures and Tables

**Table 1:** Experimental setup.

Hardware	CPU	Intel i9-12900 KF (24 cores)
	Memory	32 GB
	Storage	Samsung SSD 860 PRO 256 GB (SATA)
System	OS	Ubuntu 20.04.03 LTS with Linux kernel 5.15.21
	File system	F2FS

**Table 2:** Workload settings.

Workload-F	Records	10,000,000
	Operations	1,000,000,000
	Read: Read-modify-write	5:5
	Request distribution	zipfian

**Table 3:** Color configuration for RocksDB.

		Fragmented	Not Fragmented
RocksDB	.sst	Light orange, Dark orange	Green
	.log	Light teal, Dark teal	Blue
	Default	Black, Dark gray	Light gray

**Table 4:** Color configuration for Redis.

		Fragmented	Not Fragmented
Redis	.rdb	Light orange, Dark orange	Green
	Default	Black, Dark gray	Light gray

**Table 5:** Color configuration for VoltDB.

		Fragmented	Not Fragmented
VoltDB	.vpt	Light orange, Dark orange	Green
	.digest	Light brown, Dark brown	Green
	.hash	Light purple, Dark purple	Green
	Default	Black, Dark gray	Light gray

**Table 6:** Fio settings to simulate a heavy I/O.

Read-write	Random read/write
Job number	20
Each file size	4 MiB
Block size	4 KiB

## Data Availability

Not applicable.

## Abbreviations

The following abbreviations are used in this manuscript:

YCSB	Yahoo cloud system benchmark
OS	Operating system
RDB	Redis database
IOCTL	Input and output control
DBMS	Database management system
RDBMS	Relational database management system
ACID	Atomicity consistency isolation durability
NVMe	Non-volatile memory express
SSD	Solid state drive
RISC	Reduced instruction set computer
CXL	Compute express link
DCPMMs	Data center persistent memory modules
I/O	Input/output
F2FS	Flash-friendly file system
WAL	Write-ahead log
LBA	Logical block address
